# Chromosome band 16q24 is frequently deleted in human gastric cancer

**DOI:** 10.1038/sj.bjc.6690391

**Published:** 1999-05-01

**Authors:** Y Mori, M Matsunaga, T Abe, S Fukushige, K Miura, M Sunamura, K Shiiba, M Sato, T Nukiwa, A Horii

**Affiliations:** Departments of Molecular Pathology, andSurgery I, Tohoku University School of Medicine, Sendai 980-8575, Japan; Departments of Respiratory Oncology and Molecular Medicine, andThoracic Surgery, Institute of Development, Aging and Cancer, Tohoku University, Sendai, 980-8575, Japan

**Keywords:** gastric cancer, LOH, 16q, H-cadherin, microsatellite

## Abstract

We have analysed the loss of heterozygosity (LOH) on chromosome bands 16q22–q24 in 24 primary gastric cancer tissues and found three regions of frequent allelic loss (16q22, 16q24.1–q24.3 and 16q24.3). The region for the most frequent allelic loss (63%) was in 16q24.1–q24.3. LOH of this region had no relationship with histological subtype, but a significant association between LOH and microscopic lymphangial invasion was observed. Although not significant, vascular and gastric wall invasions are also associated with LOH. The region includes the locus for the *H-cadherin* gene. Therefore we examined the genetic and epigenetic alterations of this gene. Markedly reduced expression was observed in gastric cancer cell lines compared with that of normal gastric mucosa. However, no mutation was found in this gene in any of the gastric cancer tissues or the gastric cancer cell lines. Furthermore, we analysed the methylation status of the 5'-flanking region of the gene, but no significant association was found. We suggest that some other tumour suppresser gene(s) in 16q24.1–q24.3 may be responsible for gastric carcinogenesis. © 1999 Cancer Research Campaign


*British Journal of Cancer* (1999) **80**(3/4), 556–562
© 1999 Cancer Research Campaign
Article no. bjoc.1998.0391

### Chromosome band 16q24 is frequently deleted in human gastric cancer

**Y Mori[1,3], M Matsunaga[1], T Abe[1,2], S Fukushige[1], K Miura[2], M Sunamura[2], K Shiiba[2], M Sato[1,4], T Nukiwa[3] and A Horii[1]**

Departments of [1]Molecular Pathology and [2]Surgery I, Tohoku University School of Medicine, Sendai, 980-8575, Japan; Departments of [3]Respiratory Oncology and Molecular Medicine and [4]Thoracic Surgery, Institute of Development, Aging and Cancer, Tohoku University, Sendai, 980-8575, Japan

**Summary** We have analysed the loss of heterozygosity (LOH) on chromosome bands 16q22–q24 in 24 primary gastric cancer tissues and found three regions of frequent allelic loss (16q22, 16q24.1–q24.3 and 16q24.3). The region for the most frequent allelic loss (63%) was in 16q24.1–q24.3. LOH of this region had no relationship with histological subtype, but a significant association between LOH and microscopic lymphangial invasion was observed. Although not significant, vascular and gastric wall invasions are also associated with LOH. The region includes the locus for the *H-cadherin* gene. Therefore we examined the genetic and epigenetic alterations of this gene. Markedly reduced expression was observed in gastric cancer cell lines compared with that of normal gastric mucosa. However, no mutation was found in this gene in any of the gastric cancer tissues or the gastric cancer cell lines. Furthermore, we analysed the methylation status of the 5'-flanking region of the gene, but no significant association was found. We suggest that some other tumour suppresser gene(s) in 16q24.1–q24.3 may be responsible for gastric carcinogenesis.

**Keywords:** gastric cancer; LOH; 16q; H-cadherin; microsatellite

Gastric cancer is one of the most common cancers in the world (Parkin et al, 1988). A better understanding of its molecular mechanisms is needed to establish of effective methods for managing patients with this disease. In gastric cancers, activation of K-*ras*, c-*met*, K-*sam*, c-*erbB-2* and *EGFR* (Deng et al, 1987; Yokota et al, 1988; Hattori et al, 1990; Kameda et al, 1990; Tahara, 1990; Kuniyasu et al, 1992; Hirono et al, 1995), and inactivation of *APC*, *p53*, *DCC* and *FHIT* (Tamura et al, 1991; Horii et al, 1992; Nakatsuru et al, 1992; Barletta et al, 1993; Ohta et al, 1996) have been reported so far. Frequent loss of heterozygosity (LOH) in gastric cancer on chromosome arms 1p, 3p, 5q, 7q, 11p, 11q, 12q, 17p and 18q has suggested the involvement of mutations in several tumour suppressor genes in this disease (Sano et al, 1991; Uchino et al, 1992; Ranzani et al, 1993; Kuniyasu et al, 1994; Schneider et al, 1995; Baffa et al, 1996; Ezaki et al, 1996).

On chromosome 16, Gleeson et al (1997) reported frequent allelic losses on almost entire 16q arm in diffuse type gastric cancer, but no detailed analysis of 16q in gastric cancer has yet been done other than the studies on *E-cadherin*, which is located at 16q22.1 (Shiozaki et al, 1991; Guilford et al, 1998). Frequent LOH on 16q22–q24 have been reported in many other tumours of the liver, ovary, breast, prostate and salivary gland as well as infant brain tumours and non-small cell lung cancer (Sato et al, 1990, 1991; Nishida et al, 1992; Tsuda et al, 1994; Dorion-Bonnet et al, 1995; Iwabuchi et al, 1995; Braeker et al, 1996; Johns et al, 1996; Elo et al, 1997; Latil et al, 1997; Sato et al, 1998*a*). In this study, we analysed allelic loss on chromosome bands 16q22–q24 in 24 gastric cancer specimens and identified three regions of common allelic loss; the highest frequency of allelic loss was observed at D16S534 in 16q24.2 (63%). The *H-cadherin* gene maps to this region. This gene is a new member of the cadherin family and has been identified as a tumour suppressor gene associated with breast cancer: loss of expression has been observed immunohistochemically in invasive breast cancer tissues, and transfection of its cDNA suppressed some neoplastic phenotypes (Lee, 1996). Thus it is of great interest to investigate the expression and genetic alterations of this gene in gastric cancer.

*Received 28 July 1998*
*Revised 23 October 1998*
*Accepted 4 November 1998*

*Correspondence to:* A Horii

### MATERIALS AND METHODS

### Tissue samples and DNA extraction

Paired normal and cancerous tissues from 24 Japanese patients with gastric cancer (18 intestinal type and six diffuse type, 14 males and ten females) were obtained during surgery at Tohoku University Hospital (Sendai, Japan) and its related hospitals (Sendai, Japan) during the period from March 1995 to May 1996. Histopathological classifications were defined according to WHO criteria (1990). Clinical and pathological stages were determined by the Japanese Research Society of Gastric Cancer (JRSGC) (1981). Brief information on the clinical staging system of JRSGC was included in our previous report (Ouyang et al, 1997). None of these patients had family history for gastric cancer in first-degree relatives. In each case, tumour tissue and normal mucosa were separately collected, frozen in liquid nitrogen immediately after resection and stored at –80°C until use. DNA extraction was performed according to methods described previously (Sato et al, 1990). Details of the patients and their tumours are summarized in Table 1.

**Table 1** Characteristics of patients

| No. | Sex | Age | Histology[a] | Stage | T | N | M | Depth[b] | ly[c] | v[d] | n[e] |
|---|---|---|---|---|---|---|---|---|---|---|---|
| 25 | F | 62 | I | IA | 1 | 0 | 0 | m | 0 | 0 | 0 |
| 26 | M | 66 | I | IA | 1 | 0 | 0 | m | 0 | 0 | 0 |
| 81 | M | 61 | I | IA | 1 | 0 | 0 | sm | 0 | 0 | 0 |
| 146 | M | 78 | I | IA | 1 | 0 | 0 | sm | 0 | 0 | 0 |
| 48 | M | 68 | I | IA | 1 | 0 | 0 | sm | 1 | 0 | 0 |
| 29 | F | 53 | I | IB | 2 | 0 | 0 | mp | 1 | 0 | 0 |
| 43 | M | 68 | I | IB | 2 | 0 | 0 | mp | 1 | 0 | 0 |
| 24 | M | 66 | I | IB | 2 | 0 | 0 | mp | 1 | 2 | 0 |
| 77 | M | 73 | I | IB | 2 | 0 | 0 | mp | 2 | 1 | 0 |
| 84 | F | 68 | I | IB | 2 | 0 | 0 | ss | 0 | 0 | 0 |
| 94 | M | 50 | I | IB | 2 | 0 | 0 | ss | 1 | 0 | 0 |
| 58 | M | 64 | I | II | 3 | 0 | 0 | se | 2 | 1 | 0 |
| 67 | M | 50 | I | IIIA | 2 | 2 | 0 | ss | 0 | 0 | 2 |
| 85 | F | 63 | I | IIIA | 3 | 1 | 0 | se | 1 | 2 | 1 |
| 79 | M | 79 | I | IIIB | 3 | 2 | 0 | se | 2 | 0 | 2 |
| 97 | F | 72 | I | IV | 3 | 0 | 1 (peritoneum) | se | 2 | 1 | 0 |
| 76 | M | 74 | I | IV | 3 | 2 | 1 (liver) | se | 2 | 1 | 2 |
| 45 | F | 75 | I | IV | 4 | 2 | 0 | se | 2 | 2 | 2 |
| 73 | M | 45 | D | IA | 1 | 0 | 0 | m | 0 | 0 | 0 |
| 55 | F | 71 | D | IA | 1 | 0 | 0 | sm | 0 | 0 | 0 |
| 88 | F | 43 | D | II | 2 | 1 | 0 | ss | 1 | 0 | 1 |
| 98 | F | 55 | D | II | 3 | 0 | 0 | se | 0 | 0 | 0 |
| 78 | F | 50 | D | IIIA | 2 | 2 | 0 | ss | 2 | 1 | 2 |
| 23 | M | 74 | D | IV | 4 | 2 | 1 (peritoneum) | se | 3 | 3 | 2 |

[a]I, intestinal type of gastric cancer; D, diffuse type of gastric cancer. [b]m, mucosa; sm, submucosa; mp, muscularis propria; ss, subserosa; se, serosa exposed. [c]ly, lymphangial invasion. [d]v, vascular invasion. [e]n, nodal metastasis. M, male; F, female.

### Gastric cancer cell lines

Three cell lines, AZ521 (Imanishi et al, 1989), MKN7 (Yokota et al, 1988) and KatoIII (Nakatani et al, 1990), all derived from gastric cancer tissues, were used as sources of DNAs and RNAs. These cell lines were obtained from Cancer Cell Repository, IDAC, Tohoku University (Sendai, Japan). DNA extraction was performed as described above. RNAs were extracted as described previously (Nakamura et al, 1984).

### LOH analysis

A total of 14 microsatellite markers, listed in Figure 2, were used in the present study. Nucleotide sequence of the primers and detailed polymerase chain reaction (PCR) conditions were described previously (Sato et al, 1998*a*). Each product was run on a 3% agarose gel to check the concentration of the PCR product, diluted with a 2- to 20-fold volume of 95% formamide/0.25% methyl-violet according to its concentration, and denaturated at 80°C for 10 min. These samples were electrophoresed in 6% Long-Ranger™ (FMC Bio Products, Rockland, ME, USA)/6 M urea gels and analysed by ALFred DNA Sequencer with Fragment Manager™ software (Pharmacia Biotech, Uppsala, Sweden). We defined allelic loss as occurring when more than a 50% reduction in the area of a peak was calculated in the tumour when compared with that of corresponding normal tissue. At least two independent experiments were performed to confirm the results.

### Fluorescence in situ hybridization (FISH) analysis

Two-colour FISH was performed with cosmid clones labelled with either biotin-16-dUTP or digoxigenin-11-dUTP according to methods described previously (Fukushige et al, 1997). To eliminate background noise due to repetitive sequences, 0.15 μg μl$^{-1}$ of COT-1™ DNA (GIBCO BRL, Gaithersburg, MD, USA) was added. A centromeric plasmid probe, RMC16L007, an alpha satellite clone of chromosome 16, and a cosmid clone located at either D16S422 or D16S534 were used as probes. RMC16L007 was obtained from the Resource for Molecular Cytogenetics, LBNL/UCSF (Greig et al, 1989; Sato et al, 1998*a*). Allelic deletions were assessed by counting of signals for centromeric and cosmid probes in more than 50 nuclei.

### Analysis of expression pattern of the *H-cadherin* gene

Expression of the *H-cadherin* gene was analysed using three gastric cancer cell lines and normal gastric mucosae from two individuals by reverse transcription coupled with polymerase chain reaction (RT-PCR) as described previously (Mori et al, 1997). Expression of the *actin* gene was monitored as the control. Conditions for the PCR amplifications were as follows: 94°C for 2 min for the initial denaturation followed by 30 cycles of 94°C for 30 s, 55°C for 30 s and 72°C for 30 s. The final elongation was at 72°C for 5 min. The amplified products were electrophoresed in 3% agarose gels, transferred to nylon membranes, and hybridized with $^{32}$P end-labelled oligonucleotide probes. Nucleotide sequences of the PCR primers and the oligonucleotide probe were shown in our previous work (Sato et al, 1998*b*).

### Mutation analysis of the *H-cadherin* gene

We searched for mutations of the *H-cadherin* gene in 24 primary gastric cancer tissues and three gastric cancer cell lines. As the first screening in this search, PCR-SSCP (single-strand conformation polymorphism) was performed in all of the coding exons, as well as their surrounding regions and the 5′-flanking region as

© Cancer Research Campaign 1999

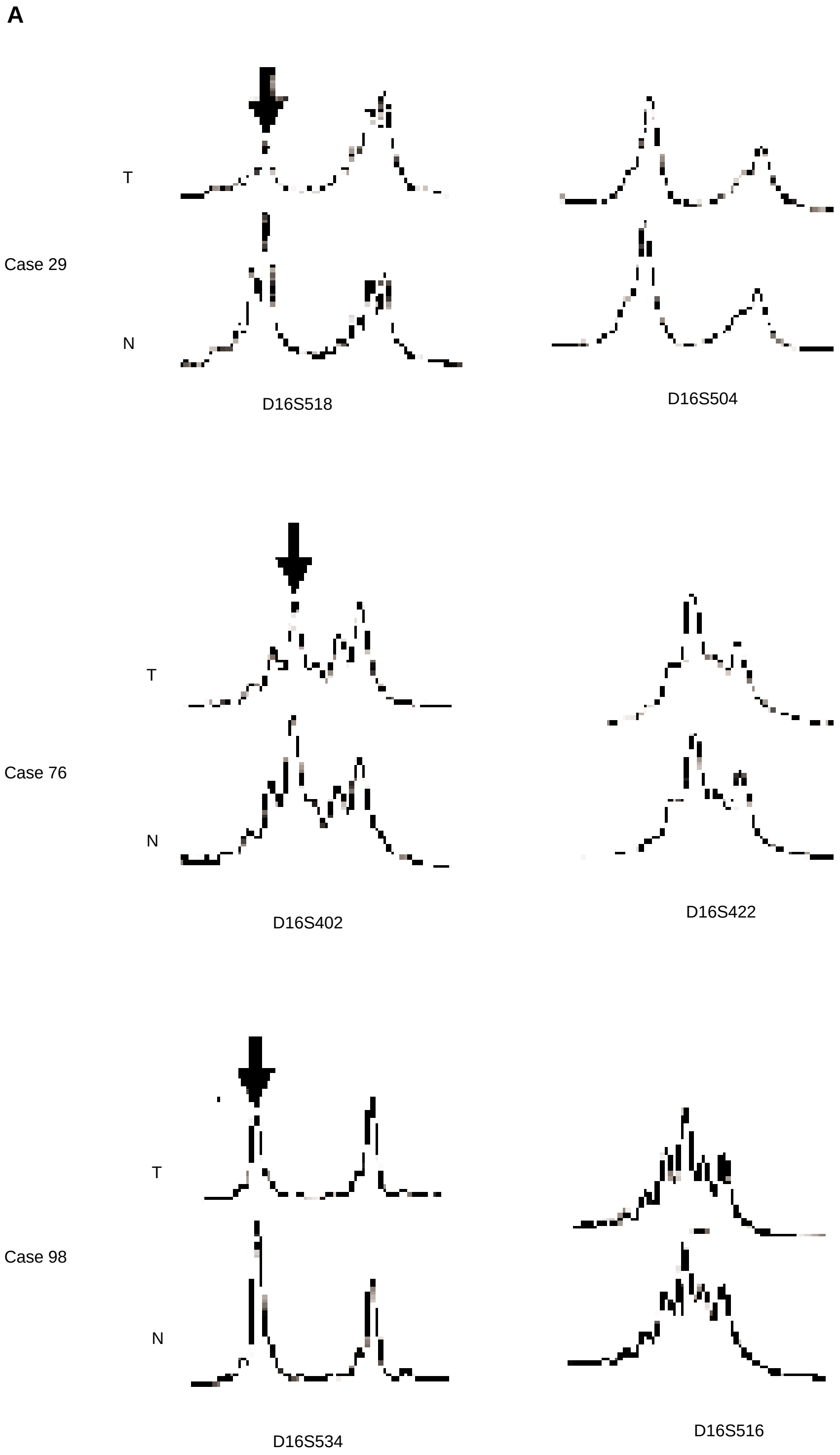

**Figure 1** Typical examples of microsatellite analyses. (**A**) Profiles of the electrophoreses of PCR products derived from tumours and their corresponding normal tissues of cases 29, 76 and 98 are shown. Loci analysed are indicated at the bottom of each set of profiles. Allelic losses are indicated by arrows: case 29 at D16S518, case 76 at D16S402 and case 98 at D16S534. Retention of heterozygosities were observed at D16S504 (case 29), D16S422 (case 76) and D16S516 (case 98). T (upper column) and N (lower column) denote DNA samples of tumour and corresponding normal tissues, respectively (*Continued*)

 © Cancer Research Campaign 1999

**B**

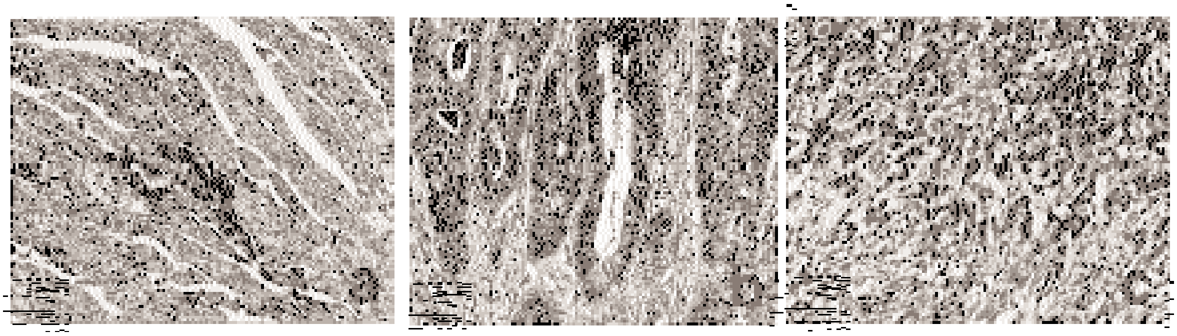

**Figure 1 Cont** (**B**) Light microscopic features of the tumours; a, case 29, intestinal type; b, case 76, intestinal type; c, case 98, diffuse type (x80, haematoxylin and eosin staining).

described by Orita et al (1989) with some modifications (Mayama et al, 1997). Aberrant bands were collected and the DNAs were purified for the second PCR amplification and sequencing.

### Analyses of methylation status of the 5′-region of the *H-cadherin* gene by methylation specific PCR

Ten primary gastric cancer tissues, three gastric cancer cell lines and two normal gastric mucosae were analysed for methylation status of the 5′ -flanking region of the *H-cadherin* gene. DNAs were modified with sodium bisulphite and analysed according to Herman’s method (1996) with some modifications (Sato et al, 1998*b*). Nucleotide sequences of the primers used in this study are: HCADMF. 5′-CGCGGGGTTCGTTTTTCGC-3’; HCADMR, 5′-GACGTTTTCATTCATACACGCG-3′; MSP, 5′-CTAATAAC-CAAAACCAATAACTTTA-3′.

### RESULTS

### Allelotype analysis

Typical examples of the microsatellite analyses are shown in Figure 1A. T and N denote PCR-amplified DNA samples of tumour and corresponding normal tissues, respectively, and the primer sets used are shown at the bottom. An allelic loss was identified at D16S518 in case 29, indicated by an arrow. Similarly, in cases 76 and 98, LOH at D16S402 and D16S534, respectively, was obvious, as indicated by arrows. Results of microsatellite analyses are summarized in Figure 2. Although the number of samples analysed was not very large, three distinct regions of common allelic loss were found: we named them as G1 through G3 in order from centromeric to telomeric. Markers and their genetic distances according to Doggett et al (1995) are also shown. We also analysed a CA dinucleotide marker in intron 1 of the *H-cadherin* gene that is between D16S422 and D16S402 (Sato et al, 1998*c*), to determine the telomeric border of G2. However, only a few cases were informative and the results did not contribute to the determination of the border of the region G2. Twelve (50%) of the 24 tumours showed allelic imbalances (AIs), presumably LOHs, at one or more loci.

Microsatellite instability (MSI) at two or more loci was observed in three (12.5%) of 24 tumours; two cases (8.3%) showed MSIs at most of the loci tested. These results are consistent with previous studies (Kuniyasu et al, 1994; Schneider et al, 1995; Ezaki et al, 1996).

Since we did not have large enough tissue volumes of these gastric cancers, we could not perform FISH or other methods with which to study whether the AIs really reflect LOHs. Instead, we analysed allelic loss at this region in three gastric cancer cell lines (AZ521, MKN7 and KatoIII) by FISH. One cell line (MKN7) exhibited an allelic loss when genomic cosmid clone harbouring D16S534 was used as the probe for FISH. MKN7 retained the alleles at both D16S402 and D16S422 loci in 16q24 (data not shown).

In this study, the locus most frequently lost was D16S534 (63%), and three common regions of allelic loss were identified: G1, an 18-cM region between D16S496 and D16S518; G2, a 3-cM region between D16S505 and D16S402; and G3, a region distal from D16S520 (see Figure 2). The incidence of LOH at D16S496, the locus for the *E-cadherin* gene, which has been reported to be inactivated in some gastric cancers of diffuse type (Shiozaki et al, 1991) as well as in some familial gastric cancers (Guilford et al, 1998), was not very high (2/5 in diffuse type and 3/10 in intestinal type).

**Table 2** Characteristics of patients with and without LOH in region G2

| | | LOH + | LOH – | |
|---|---|---|---|---|
| Age | | | | |
| 60≤ | | 8 | 6 | *P* = 0.574 |
| 60> | | 3 | 3 | |
| Sex | | | | |
| M | | 8 | 5 | *P* = 0.370 |
| F | | 3 | 4 | |
| Histologic subtype | | | | |
| I[a] | | 8 | 6 | *P* = 0.574 |
| D[a] | | 3 | 3 | |
| Pathologic features | | | | |
| ly[b] | – | 1 | 5 | *P* = 0.038 |
| | + | 10 | 4 | |
| v[b] | – | 4 | 7 | *P* = 0.080 |
| | + | 7 | 2 | |
| n[b] | – | 8 | 4 | *P* = 0.205 |
| | + | 3 | 5 | |
| Depth | | | | |
| ~sm[b] | | 1 | 4 | *P* = 0.098 |
| mp~[b] | | 10 | 5 | |

[a]I, interstitial type of gastric cancer; D, diffuse type of gastric cancer. [b]ly, lymphangial invasion; v, vascular invasion; n, nodal metastasis; sm, submucosa; mp, muscularis propria. M, male; F, female.

*© Cancer Research Campaign 1999*

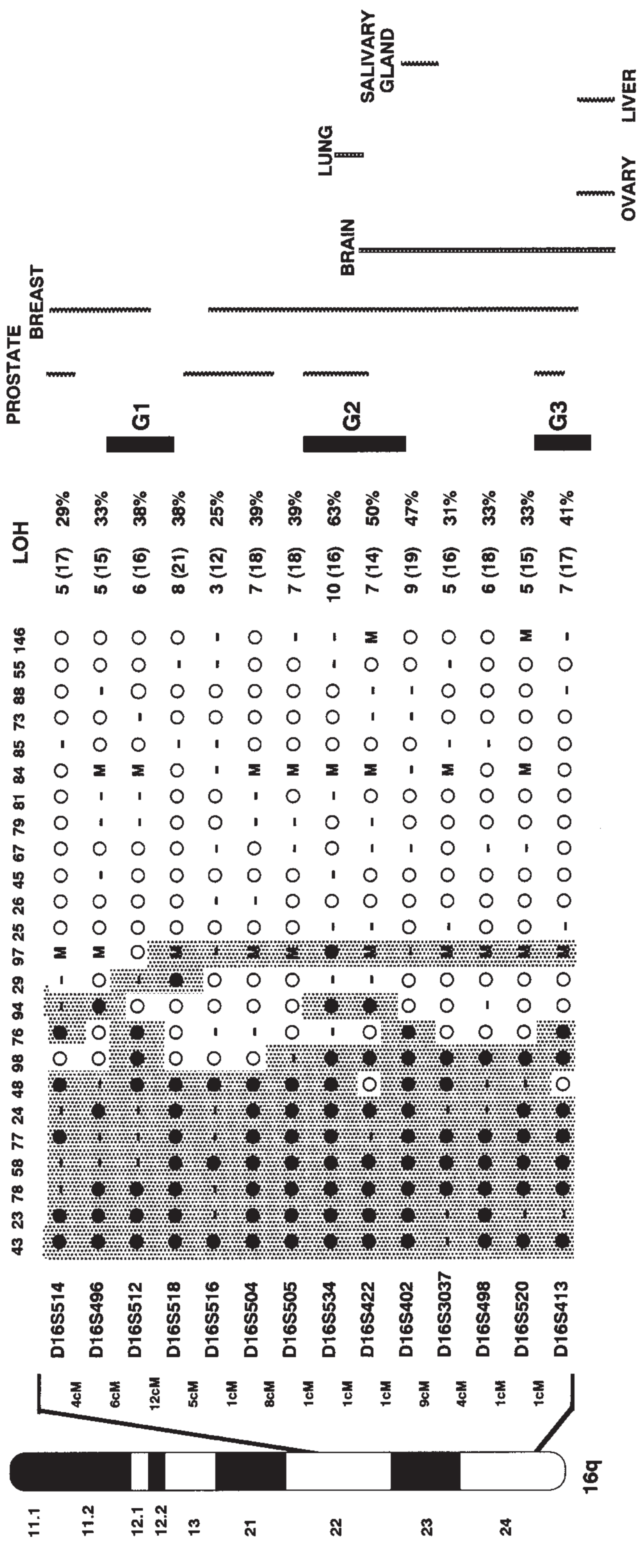

**Figure 2** Results of the microsatellite analysis. (○), retention of heterozygosity; (●), loss of heterozygosity (LOH); –, not informative; M, microsatellite instability (MSI). Hatched boxes also indicate the lost regions. G1, G2, and G3 denote regions of common allelic loss. Regions previously reported to be frequently lost in each type of tumour are also shown on the right. In most of the cases, markers used in studies by others were different from ours. Relative location of the deleted region was indicated by each bar: prostate cancer (Elo et al, 1997; Latil et al, 1997); breast cancer (Sato et al, 1991; Tsuda et al, 1994; Dorion-Bonnet et al, 1995); brain tumour (Braeker et al, 1996); lung cancer (Sato et al, 1998*a*); salivary gland tumour (Johns et al, 1996); hepatocellular carcinoma (Nishida et al, 1992); ovarian cancer (Sato et al, 1990; Iwabuchi et al, 1995).

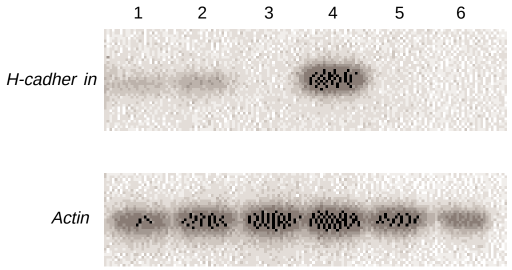

**Figure 3** Results of RT-PCR analysis of gastric cancer cell lines and normal gastric mucosae. Lanes 1 and 2: normal gastric mucosae derived from different individuals; lanes 3, 5 and 6: gastric cancer cell lines AZ521, KatoIII and MKN7; lane 4: a mixture of *actin* and *H-cadherin* cDNA clones as the positive control.

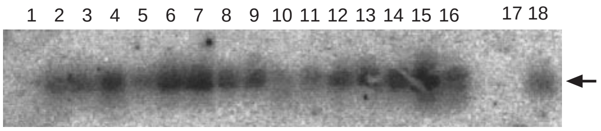

**Figure 4** Results of methylation-specific PCR analysis of the 5′-flanking region of the *H-cadherin* gene using methylated-DNA-specific primers. An arrow indicates signals derived from the methylated allele. Lane 1: negative control (water); lanes 2 and 3: normal gastric mucosae; lanes 4–13: gastric cancer tissues (cases 29, 45, 67, 76, 79, 81, 84, 85, 94 and 98); lanes 14–16: gastric cancer cell lines (MKN7, AZ521 and KatoIII); lane 17: unmodified genomic DNA extracted from the peripheral blood cells of a normal volunteer; lane 18: positive control.

Clinicopathologic features of the cases that showed LOHs in G2 are summarized in Table 2. Statistical analysis was done by Fisher's exact test. LOHs at this region did not significantly correlate with age, sex, or histologic subtype. Cases that showed LOHs at G2 correlated with lymphangial invasion ($P = 0.038$). Although it was not significant, G2 LOH tended to be high in tumours with vascular invasion as well as invasion into the gastric wall.

### Analysis for the alterations of the *H-cadherin* gene in gastric cancer

One of the putative tumour suppressor genes located at G2 was *H-cadherin*, a newly isolated adhesion molecule in the cadherin family. This gene has been reported to have relationships with tumour cell invasion in breast cancers (Lee, 1996). Thus, it is one candidate for a tumour suppressor gene for gastric cancers. We next analysed the expression and genetic alterations of this gene.

RT-PCR was performed to determine the expression of *H-cadherin* in normal gastric mucosae from two individuals as well as in three gastric cancer cell lines (AZ521, MKN7 and KatoIII). A decrease in *H-cadherin* expression was observed in all three gastric cancer cell lines when compared to normal gastric tissue (Figure 3). Northern hybridization was also performed, and we confirmed the results of RT-PCR (data not shown).

We then searched for genetic alterations in the entire coding region as well as for exon–intron boundaries of the *H-cadherin* gene. PCR-SSCP and genomic DNA sequencing were used for detection of mutations, but none were observed.

We further analysed the methylation status of the 5′-flanking region of *H-cadherin* (nucleotide position from –265 to –25) in ten gastric cancer tissues and the three gastric cancer cell lines

 *© Cancer Research Campaign 1999*

(AZ521, MKN7 and KatoIII), as well as two normal gastric mucosae. Since there was no restriction site suitable for detection of methylation status in this gene, we performed methylation specific PCR analysis (Herman et al, 1996). All of the tumour samples and normal tissues had methylated alleles (Figure 4).

### DISCUSSION

In this study, we analysed 16q22–q24 and observed frequent allelic loss (63% at maximum): three commonly deleted regions were identified, although the number of tumours analysed was not large. LOH of these regions was associated with microscopic invasion, especially that to the lymphangial tract. Gleeson et al (1997) reported that LOH on 16q is more frequently observed in gastric cancers of the diffuse type (78%) than those of the intestinal type (27%). Our results, however, found no significant difference between them. The differences may be accounted for the limited number of tumours analysed in these studies. Alternatively, different genetic backgrounds and/or carcinogens due to different environmental conditions and/or lifestyles may cause these differences.

Frequent allelic losses in the 16q arm have also been reported in invasive breast cancers and metastatic prostate cancers. It has been suggested that inactivation of the same tumour suppressor gene in this region may play a role in these types of cancers, resulting in invasion and/or metastasis.

The G2 region was the locus of the most frequent loss in this study (63% at D16S534). This high frequency of allelic loss suggested the localization of a tumour suppressor gene that plays an important role in the genesis and/or progression of gastric cancer. Allelic losses of the G2 region are also reported in cancers of the breast and prostate, as well as in mucoepidermoid tumour of the salivary gland and infant brain tumour. More than ten expressed sequence tags (ESTs) map to this region. Among these, *H-cadherin*, *17β-hydroxysteroid dehydrogenase type II*, *transcription factor SL*-1, and *phosphatidylinositol-4,5-biphosphate phosphodiesterase* $\gamma 2$ are identified to some extent. In this study, we extensively analysed the *H-cadherin* gene, an adhesion molecule. We selected this gene because it was localized in the G2 region and included D16S534 and D16S422 within its introns (Sato et al, 1998*b*). Although all of the three gastric cancer cell lines examined expressed markedly reduced levels of *H-cadherin* mRNA, no mutation or difference in methylation status of the 5′ non-coding region was found. Perhaps a *cis*-acting element(s) of this gene lies more upstream than the region examined. Alternatively, the primary lesion could be a *trans*-acting element(s) of the *H-cadherin* gene. It would be of great interest to investigate whether or not demethylation of the *H-cadherin* gene has an effect on gastric cell proliferation. There is another possibility that some tumour suppressor gene(s) other than the *H-cadherin* gene may remain undiscovered in this region.

Two other regions were also identified as loci of frequent allelic loss. The G1 region (between D16S496 and D16S518) overlapped those of frequent allelic loss in cancers of the breast and prostate. Chromosomal rearrangement involving 16q is observed frequently in acute myelogenous leukaemia (AML) and some prostate cancer cell lines. The break point for both tumour types is located in 16q22, and the *core binding factor* $\beta$ and the *hpr* genes are involved in AML and prostate cancer, respectively (Shurtleff et al, 1995; Veronese et al, 1996).

A previous report showed that the *E-cadherin* gene was inactivated in some diffuse-type gastric cancers. Moreover, this gene was found to be responsible for some familial gastric cancers. According to our results, this gene was localized at the flank of the region G1. It has been suggested that mutations of the *E-cadherin* gene might associate with a subset of sporadic gastric cancers. Region G3 (distal from D16S520) also overlapped those of frequent allelic loss in cancers of the breast, prostate, ovary and liver. However, region G3 is localized very close to the telomere of chromosome 16q, and the LOH at this region might merely be the result of non-specific chromosome deletion with little, if any, biological significance in gastric carcinogenesis.

### ACKNOWLEDGEMENTS

The authors are grateful to Dr Akira Sakurada (Tohoku University) for his help in analysis of methylation status. This work was supported by the Ministry of Education, Science, Sports and Culture of Japan and Vehicle Racing Commemorative Foundation.

### REFERENCES

Baffa R, Negrini M, Mandes B, Rugge M, Ranzani GN, Hirohashi S and Croce CM (1996) Loss of heterozygosity for chromosome 11 in adenocarcinoma of the stomach. *Cancer Res* **56**: 268–272

Barletta C, Scillato F, Sega FM and Mannella E (1993) Genetic alteration in gastrointestinal cancer. A molecular and cytogenetic study. *Anticancer Res* **13**: 2325–2329

Braeker H, Rashee ABK, McLendon RE, Friedman HS, Batra SK, Fuchs HE and Binger SH (1996) Microsatellite analysis of childhood brain tumor. *Genes Chromosomes Cancer* **15**: 54–63

Deng G, Lu Y, Chen S, Miao J, Lu G, Li H, Cai H, Xu X EZ and Liu P (1987) Activated c-Ha-*ras* oncogene with a guanine to thymine transversion at the twelfth codon in a human stomach cancer cell line. *Cancer Res* **47**: 3195–3198

Doggett NA, Goodwin LA, Tesmer JG, Meincke LJ, Bruce DC, Clark LM, Altherr MR, Ford AA, Chi H-C, Marrone BL, Longmire JL, Lane SA, Whitmore SA, Lowenstein MG, Sutherland RD, Mundt MO, Knill EH, Bruno WJ, Macken CA, Torney DC, Wu JR, Griffith J, Sutherland GR, Deaven LL, Callen DF and Moyzis RK (1995) An integrated physical map of human chromosome 16. *Nature* **377**: 335–366

Dorion-Bonnet F, Mautalen S, Hostein I and Longy M (1995) Allelic imbalance study of 16q in human primary breast carcinomas using microsatellite markers. *Genes Chromosomes Cancer* **14**: 171–181

Elo JP, Härkonen P, Kyllönen AP, Lukkarinen O, Poutanen M, Vihko R and Vihko P (1997) Loss of heterozygosity at 16q24.1–q24.2 is significantly associated with metastatic and aggressive behavior of prostate cancer. *Cancer Res* **57**: 3356–3359

Ezaki T, Yanagisawa A, Ohta K, Aiso S, Watanabe M, Hibi T, Kato Y, Nakajima T, Ariyama T, Inazawa J, Nakamura Y and Horii A (1996) Deletion mapping on chromosome 1p in well-differentiated gastric cancer. *Br J Cancer* **73**: 424–428

Fukushige S, Waldman FM, Kimura M, Abe T, Furukawa T, Sunamura M, Kobari M and Horii A (1997). Frequent gain of copy number on the long arm of chromosome 20 in human pancreatic adenocarcinoma. *Genes Chromosomes Cancer* **19**: 161–169

Gleeson CM, Sloan JM, McGuigan JA, Ritchie AJ, Weber JL and Russell SEH (1997) Allelotype analysis of adenocarcinoma of the gastric cardia. *Br J Cancer* **76**: 1455–1465

Guilford P, Hopkins J, Harraway J, McLeod M, McLeod N, Harawira P, Taite H, Scoular R, Miller A and Reeve AE (1998) E-cadherin germline mutations in familial gastric cancer. *Nature* **392**: 402–404

Greig GM, England SB, Bedford HM and Willard HF (1989) Chromosome-specific alpha satellite DNA from the centromere of human chromosome 16. *Am J Hum Genet* **45**: 862–872

Hattori Y, Odagiri H, Nakatani H, Miyagawa K, Naito K, Sakamoto H, Katoh O, Yoshida T, Sugimura T and Terada M (1990) K-*sam*, an amplified gene in stomach cancer, is a member of the heparin-binding growth factor receptor genes. *Proc Natl Acad Sci USA* **87**: 5983–5987

© Cancer Research Campaign 1999

Herman JG, Graff JR, Myöhänen S, Nelkin BD and Baylin SB (1996) Methylation-specific PCR: a novel PCR assay for methylation status of CpG islands. *Proc Natl Acad Sci USA* **93**: 9821–9826

Hirono Y, Tsugawa K, Fushida S, Ninomiya I, Yonemura Y, Miyazaki I, Endou Y, Tanaka M and Sasaki T (1995) Amplification of epidermal growth factor receptor gene and its relationship to survival in human gastric cancer. *Oncology* **52**: 182–188

Horii A, Nakatsuru S, Miyoshi Y, Ichii S, Nagase H, Kato Y, Yanagisawa A and Nakamura Y (1992) The *APC* gene, responsible for familial adenomatous polyposis, is mutated in human gastric cancer. *Cancer Res* **52**: 3231–3233

Imanishi K, Yamaguchi K, Suzuki M, Honda S, Yanaihara N and Abe K (1989) Production of transforming growth factor-β in human gastric cancer cell lines. *Br J Cancer* **59**: 761–765

Iwabuchi H, Sakamoto M, Sakunaga H, Ma YY, Carcangiu ML, Pinkel D, Yang-Feng TL and Gray JW (1995) Genetic analysis of benign, low-grade, and high-grade ovarian tumors. *Cancer Res* **55**: 6172–6180

Japanese Research Society For Gastric Cancer (1981) The general rules for the gastric cancer study in surgery and pathology. *Jpn J Surg* **11**: 127–139

Johns MM III, Westra WH, Califano JA, Eisele D, Koch WM and Sidransky D (1996) Allelotype of salivary grand tumors. *Cancer Res* **56**: 1151–1154

Kameda T, Yasui W, Yoshida K, Tsujino T, Nakayama H, Ito M, Ito H and Tahara E (1990) Expression of ERBB2 in human gastric carcinomas: relationship between $p185^{ERBB2}$ expression and the gene amplification. *Cancer Res* **50**: 8002–8009

Kuniyasu H, Yasui W, Kitadai Y, Yokozaki H, Ito H and Tahara E (1992) Frequent amplification of the c-*met* gene in scirrhous type stomach cancer. *Biochem Biophys Res Commun* **189**: 227–232

Kuniyasu H, Yasui W, Yokozaki H, Akagi M, Akama Y, Kitahara K, Fujii K and Tahara E (1994) Frequent loss of heterozygosity of the long arm of chromosome 7 is closely associated with progression of human gastric carcinomas. *Int J Cancer* **59**: 597–600

Latil A, Cussenot O, Fournier G, Driouch K and Lidereau R (1997) Loss of heterozygosity at chromosome 16q in prostate adenocarcinoma: Identification of three independent regions. *Cancer Res* **57**: 1058–1062

Lee SW (1996) *H-cadherin*, a novel cadherin with growth inhibitory functions and diminished expression in human breast cancer. *Nature Med* **2**: 776–782

Mayama T, Mori T, Abe K, Sasano H, Nishihira T and Horii A (1997) Analysis of the p53 gene mutations in patients with multiple primary cancers of the esophagus. *Eur J Surg Oncol* **23**: 298–303

Mori Y, Shiwaku H, Fukushige S, Wakatsuki S, Sato M, Nukiwa T and Horii A (1997) Alternative splicing of *hMSH2* in normal human tissues. *Hum Genet* **99**: 590–595

Nakamura Y, Ogawa M, Nishide T, Emi M, Kosaki G, Himeno S and Matsubara K (1984) Sequences of cDNAs for human salivary and pancreatic α-amylases. *Gene* **28**: 263–270

Nakatani H, Sakamoto H, Yoshida T, Yokota J, Tahara E, Sugimura T and Terada M (1990) Isolation of an amplified DNA sequence in stomach cancer. *Jpn J Cancer Res* **81**: 707–710

Nakatsuru S, Yanagisawa A, Ichii S, Tahara E, Kato Y, Nakamura Y and Horii A (1992) Somatic mutation of the APC gene in gastric cancer: frequent mutations in very well differentiated adenocarcinoma and signet-ring cell carcinoma. *Hum Mol Genet* **1**: 559–563

Nishida N, Fukuda Y, Kokuryu H, Sadamoto T, Isowa G, Honda K, Yamanaka Y, Ikenega M, Imura H and Ishizaki K (1992) Accumulation of allelic loss on arms of chromosomes 13q, 16q, and 17p in the advanced stages of human hepatocellular carcinoma. *Int J Cancer* **51**: 862–868

Ohta M, Inoue H, Cotticelli MG, Kastury K, Baffa R, Palazzo J, Siprashvili Z, Mori M, McCue P, Druck T, Croce CM and Huebner K (1996) The *FHIT* gene, spanning the chromosome 3p14.2 fragile site and renal carcinoma-associated t(3;8) breakpoint, is abnormal in digestive tract cancers. *Cell* **84**: 587–597

Orita M, Suzuki Y, Sekiya T and Hayashi K (1989) Rapid and sensitive detection of point mutations and DNA polymorphisms using the polymerase chain reaction. *Genomics* **5**: 874–879

Ouyang H, Shiwaku HO, Hagiwara H, Miura K, Abe T, Kato Y, Ohtani H, Shiiba K, Souza RF, Meltzer SJ and Horii A (1997) The *insulin-like growth factor II receptor* gene is mutated in genetically unstable cancers of the endometrium, stomach and colorectum. *Cancer Res* **57**: 1851–1854

Parkin DM, Laara E and Muir CS (1988) Estimates of the worldwide frequency of sixteen major cancers in 1980. *Int J Cancer* **41**: 184–197

Ranzani GN, Renault B, Pellegata NS, Fattorini P, Magni E, Bacci F and Amadori D (1993) Loss of heterozygosity and K-ras gene mutations in gastric cancer. *Hum Genet* **92**: 244–249

Sano T, Tsujino T, Yoshida K, Nakayama H, Haruma K, Ito H, Nakamura Y, Kajiyama G and Tahara E (1991) Frequent loss of heterozygosity on chromosomes 1q, 5q, and 17p in human gastric carcinomas. *Cancer Res* **51**: 2926–2931

Sato M, Mori Y, Sakurada A, Fukushige S, Ishikawa Y, Tsuchiya E, Saito Y, Nukiwa Y, Fujimura S and Horii A (1998*a*) Identification of a 910 kb region of common allelic loss in chromosome bands 16q24.1–q24.2 in human lung cancer. *Genes Chromosomes Cancer* **22**: 1–8

Sato M, Mori Y, Sakurada A, Fujimura S and Horii A (1998*b*) The *H-cadherin* (*CDH13*) gene is inactivated in human lung cancer. *Hum Genet* **103**: 96–101

Sato M, Mori Y, Sakurada A, Fujimura S and Horii A (1998*c*) A GT dinucleotide repeat polymorphism in intron 1 of the *H-cadherin* (*CDH13*) gene. *J Hum Genet* **43**: 285–286

Sato T, Saito H, Morita R, Koi S, Lee JH and Nakamura Y (1991) Allelotype of human ovarian cancer. *Cancer Res* **51**: 5118–5122

Sato T, Tanigami A, Yamakawa K, Akiyama F, Kasumi F, Sakamoto G and Nakamura Y (1990) Allelotype of breast cancer: cumulative allele losses promote tumor progression in primary breast cancer. *Cancer Res* **50**: 7184–7189

Schneider BG, Pulitzer DR, Brown RD, Prihoda TJ, Bostwick DG, Saldivar V, Rodriguez-Martinez HA, Gutiérrez-Diaz CME and O'Connell P (1995) Allelic imbalance in gastric cancer: an affected site on chromosome arm 3p. *Genes Chromosomes Cancer* **13**: 263–271

Shiozaki H, Tahara H, Oka H, Miyata M, Kobayashi K, Tamura S, Iihara K, Doki Y, Hirano S, Takeichi M and Mori T (1991) Expression of immunoreactive E-cadherin adhesion molecules in human cancers. *Am J Pathol* **139**: 17–23

Shurtleff SA, Mayers S, Hiebert SW, Raimondi SC, Head DR, William CL, Wolman S, Slovak ML, Carroll AJ, Behm F, Hulshof MG, Motroni TA, Okuda T, Liu P, Collins FS and Downing JR (1995) Heterogeneity in *CBFβ/MYH11* fusion messages encoded by the inv(16)(p13q22) and the t(16;16)(p13;q22) in acute myelogenous leukemia. *Blood* **85**: 3695–3703

Tahara E (1990) Growth factors and oncogenes in human gastrointestinal carcinomas. *J Cancer Res Clin Oncol* **116**: 121–131

Tamura G, Kihana T, Nomura K, Terada M, Sugimura T and Hirohashi S (1991) Detection of frequent p53 gene mutations in primary gastric cancer by cell sorting and polymerase chain reaction single -strand conformation polymorphism analysis. *Cancer Res* **51**: 3056–3058

Tsuda H, Callen DF, Fukutomi T, Nakamura Y and Hirohashi S (1994) Allele loss on chromosome 16q24.2-qter occurs frequently in breast cancers irrespectively of differences in phenotype and extent of spread. *Cancer Res* **54**: 513–517

Uchino S, Tsuda H, Noguchi M, Yokota J, Terada M, Saito T, Kobayashi M, Sugimura T and Hirohashi S (1992) Frequent loss of heterozygosity at the DCC locus in gastric cancer. *Cancer Res* **52**: 3099–3102

Veronese ML, Bullrich F, Negrini M and Croce CM (1996) The t(6;16)(p21;q22) chromosome translocation in the LNCaP prostate carcinoma cell line results in a *tpc/hpr* fusion gene. *Cancer Res* **56**: 728–732

WHO (1990) *Histopathological Typing of Oesophageal and Gastric Tumors*, 2nd edn, pp. 20–26. Springer-Verlag: Heidelberg

Yokota J, Yamamoto T, Miyajima N, Toyoshima K, Nomura N, Sakamoto H, Yoshida T, Terada M and Sugimura T (1988) Genetic alterations of the *c-erbB-2* oncogene occur frequently in tubular adenocarcinoma of the stomach and are often accompanied by amplification of the v-*erbA* homologue. *Oncogene* **2**: 283–287

 *© Cancer Research Campaign 1999*
